# Prevalence and Risk Factors of Ovine Fasciolosis in Sheep Slaughtered at Addis Ababa Abattoir Enterprise, Ethiopia

**DOI:** 10.1155/vmi/1624127

**Published:** 2026-05-25

**Authors:** Teshager Dubie, Mimi Kalilo, Gebremedhin Gebrezgabiher

**Affiliations:** ^1^ Department of Veterinary Medicine, College of Veterinary Medicine and Animal Sciences, Samara University, P.O. Box 132, Samara, Ethiopia, su.edu.et

**Keywords:** Abattoir, Addis Ababa, Ethiopia, *Fasciola hepatica*, fasciolosis, liver condemnation, prevalence, risk factor, sheep

## Abstract

Ovine fasciolosis is a widely prevalent trematode disease of small ruminants in Ethiopia that causes significant animal health and economic impacts, particularly in sheep. A cross‐sectional study was carried out at Addis Ababa Abattoir Enterprise, Ethiopia, from December 2022 to May 2023 with the objective of estimating the prevalence of ovine fasciolosis and assessing associated risk factors. A total of 384 sheep were sampled and subjected to antemortem and postmortem examinations. Descriptive statistics, chi‐square (*X* ^2^) tests, and a multivariable logistic regression model were employed for data analysis, with statistical significance set at *p* < 0.05 and 95% confidence intervals (CI). Out of 384 sheep examined, the overall prevalence of ovine fasciolosis was estimated to be 20.57% (*n* = 79/384; 95% CI: 16.53%–24.61%). The present study identified breed (*p* < 0.001), body condition score (*p* = 0.001), and the geographical origin of sheep (*p* < 0.001) as associated risk factors affecting the prevalence of ovine fasciolosis. Local breed sheep had significantly higher odds of ovine fasciolosis infection than crossbreeds, being 3.27 times more likely to be affected (AOR = 3.27; 95% CI: 1.79–5.98; *p* < 0.001). Sheep with poor body condition (32.11%) were significantly more likely to be infected with ovine fasciolosis than those in good body condition (AOR = 3.70; 95% CI: 1.89–7.25; *p* < 0.001). In addition, sheep from Debre Berhan had significantly higher odds (AOR = 5.31; 95% CI: 2.11–13.36), while those from Bishoftu showed lower odds (AOR = 0.39; 95% CI: 0.16–0.97). In conclusion, ovine fasciolosis remains prevalent among sheep slaughtered at the study abattoir. Breed, body condition, and geographic origin were significant risk factors. Control of fasciolosis should emphasize strategic deworming in high‐risk areas, improved husbandry, farmer education programs, routine surveillance, and ongoing epidemiological research.

## 1. Introduction

Gastrointestinal helminth infections are major parasitic diseases that negatively affect livestock production efforts in Ethiopia. Among parasitic diseases of small ruminants, fasciolosis is one of the most important in Ethiopia [1]. This disease is caused by trematodes from the genus *Fasciola*, commonly known as liver flukes. Fasciolosis leads to substantial economic losses and health problems within the livestock industry across many countries or worldwide [2, 3]. *Fasciola hepatica (F. hepatica) and Fasciola gigantica (F. gigantica)* are the two species of greatest veterinary importance, with Lymnaeid freshwater snails (family Lymnaeidae) serving as their intermediate hosts. In Ethiopia, both *F. hepatica* and *F. gigantica* have been confirmed. These parasites are known to cause significant morbidity and mortality in small ruminants, leading to conditions such as anemia, hypoproteinemia, and weight loss [4–6].

Clinically, fasciolosis is marked by lethargy, weakness, anorexia, and a progressively deteriorating condition accompanied by anemia and hypoalbuminemia, which may result in ascites, pale mucous membranes, emaciation, and submandibular edema [7, 8]. Subacute fasciolosis can be triggered by ingestion of a moderate number of metacercariae, leading to anemia, jaundice, and poor body condition. Migrating flukes cause hepatic hemorrhage and tissue destruction, which may lead to anemia, liver failure, and death within 8–10 weeks [8]. Chronic fasciolosis develops several weeks after the acute phase, occurring once the parasite reaches the hepatic bile duct [2, 9]. Chronic fasciolosis occurs mainly in summer and autumn but may develop year‐round when sheep graze on heavily contaminated pastures. Infected animals shed *Fasciola* eggs in feces, which hatch into miracidia in water and infect snails. The snails release cercariae that encyst as metacercariae on aquatic plants. Cattle and sheep become infected by grazing on contaminated vegetation and humans by eating raw aquatic plants such as watercress. Proper cooking prevents infection, but raw consumption increases human fasciolosis risk in endemic areas [10, 11]. Ovine fasciolosis is an important health threat in Ethiopia, affecting sheep and resulting in substantial economic losses [12]. It is estimated that Ethiopia incurs annual losses of approximately 48.4 million birr due to ovine fasciolosis, with mortality, production loss, and liver condemnation accounting for 46.5%, 48.8%, and 4.7% losses, respectively [6]. Although numerous studies have investigated the prevalence and economic importance of sheep fasciolosis in Ethiopia, no recent study has estimated ovine fasciolosis prevalence at the Addis Ababa Abattoir Enterprise, despite its central role in meat supply for the capital [13]. Therefore, the objective of this study was to estimate the prevalence of ovine fasciolosis and assess associated risk factors at the Addis Ababa Abattoir Enterprise, Ethiopia.

## 2. Materials and Methods

### 2.1. Description of the Study Area

**FIGURE 1:**
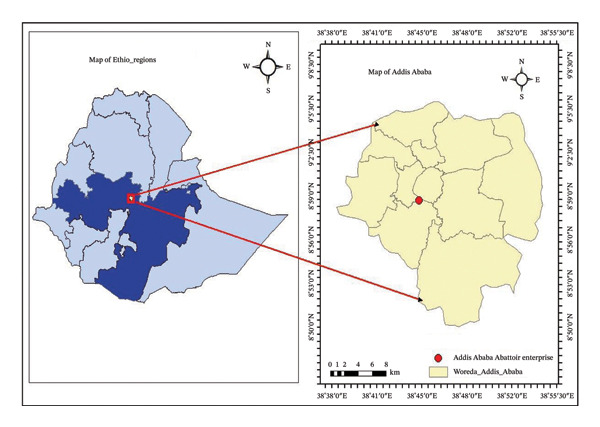
Map demonstrating the Addis Ababa Abattoir enterprise (source: ArcGIS).

The study was conducted at Addis Ababa Abattoir Enterprise, the largest slaughter facility in the city, processing cattle, small ruminants, and swine for local consumption (Figure 1). Addis Ababa is located in the central highlands of Ethiopia at an altitude of 2500 m above sea level. The average annual temperature and rainfall of the city are 21°C and 1800 mm, respectively. The relative humidity varies from 70% to 80% during the rainy season and from 40% to 50% during the dry season. The rainy season creates favorable environmental conditions for the survival and multiplication of lymnaeid snails, which are the intermediate hosts of ovine fasciolosis. An average of 183,000 cattle, 42,200 sheep, 4700 goats, and 830 pigs are slaughtered annually in the abattoir [14]. In general, the sheep slaughtered in the abattoir come from different regions and agroecological zones of the country.

### 2.2. Study Design and Period

A cross‐sectional study design was employed from December 2022 to May 2023 to estimate the prevalence of ovine fasciolosis and to assess its associated risk factors in sheep slaughtered at Addis Ababa Abattoir Enterprise, Ethiopia.

### 2.3. Sample Size Determination and Sampling Techniques

Animals were selected using a systematic sampling method. To determine the sample size, an expected prevalence of 50% was taken into consideration due to the absence of previous studies on ovine fasciolosis in the area. The desired sample size for this study was calculated using the formula given by Thrusfield [15] with a 95% confidence interval (CI) and 5% absolute precision. The sample size was calculated as follows:

(1) n=Z2x Pexp1−Pexpd2,

where *n* = required sample size. *P*_exp_ = expected prevalence. *d* = desired absolute precision (usually 0.05). 1.96 = *Z*‐value for 95% confidence level.

Based on the given formula, considering an expected prevalence of 50% with a 95% confidence level and an absolute precision of 5%, a total of 384 sheep were sampled in this study.

### 2.4. Study Animals

The study animals comprised sheep slaughtered at the Addis Ababa Abattoir Enterprise. These animals originated from six different localities: Sululta, Debre Berhan, Wolaita Sodo, Bishoftu, Arba Minch and Arsi. Animals of different age groups and both sexes were included in the study. The study populations consisted of local breeds and crossbreeds categorized into different age groups: young sheep (less than 1 year old) and adult sheep (above 1 year old). Age determination of the study animals was performed based on eruption patterns of permanent incisor teeth as described by Desta [16]. In addition, the body condition of the study animals was assessed using a standardized scoring system ranging from 0 to 5 [17], where score 0 indicates the poorest condition and score 5 the best. For analysis, sheep were categorized into three groups: poor (scores 0–1), medium (scores 2–3), and good (scores 4–5).

### 2.5. Antemortem Inspection

The antemortem examination was conducted in a well‐lit area, allowing for individual and collective assessment of the animals at rest and in motion. Details concerning the animals’ breed, age, origin, and physical condition were gathered.

### 2.6. Postmortem Inspection

During meat inspection, the livers of the previously identified animals were carefully examined for the presence of *Fasciola* species. Fluke recovery followed the procedure described by Urquhart et al. [18]. In brief, each liver was sliced into approximately 1 cm thick sections and placed in a metal trough containing warm water to allow mature flukes lodged in the smaller bile ducts to emerge. The gallbladder was also removed and washed to recover any mature flukes. Species identification was conducted based on morphological features including size, cephalic cone shape, shape of shoulders, and posterior tapering and categorized as *F. hepatica* (relatively small), *F. gigantica* (relatively large and more leaf‐like), and unidentified, as the parasites are immature [18, 19].

### 2.7. Economic Loss Due to Liver Condemnation

The total annual financial loss incurred due to liver condemnation at the Addis Ababa Enterprise abattoir was computed by multiplying the average number of sheep slaughtered annually in the abattoir with the prevalence of fasciolosis obtained from the present finding and the current mean price of the liver in the town by adapting the formula of [20] as follows:

(2) EL=Srx∗Coy∗Roz,

where EL: estimated annual economic loss due to organ condemnation; Srx: annual sheep slaughter rate of the abattoir; Coy: average cost of each sheep liver; and Roz: condemnation rates of sheep liver. The current mean price of the liver in the town was determined based on the abattoir records of the enterprise.

### 2.8. Data Management and Analysis

The collected data were entered and stored into a Microsoft Excel spreadsheet. The data were thoroughly screened for errors and properly coded before being subjected to statistical analysis. The data were imported from Microsoft Excel and analyzed using Statistical Package for Social Sciences (SPSS) software version 20. Descriptive statistics were used to estimate the prevalence of ovine fasciolosis while a Pearson chi‐square (*X* ^2^) test was used to evaluate the associations of different variables with the prevalence of ovine fasciolosis. In all calculations, the CI and the significance level were set at 95% and 5%, respectively.

## 3. Results

### 3.1. Prevalence of Ovine Fasciolosis

In the present study, 384 sheep were examined, and the overall prevalence of ovine fasciolosis was estimated to be 20.57% (*n* = 79/384; 95% CI: 16.53%–24.61%) based on postmortem examination in the Addis Ababa Abattoir Enterprise during the study period. Of the animals assessed, 240 (62.5%) were female, 230 (59.9%) were adults, 220 (57.29%) were local breeds, and 150 (39.06%) had good body condition. The demographic characteristics of the study population are presented in Table 1.

**TABLE 1:** The demographic characteristics of the sampled animals in the study area.

No	Variables	Category	No of examined animals	Percentage (%)
1	Age	Adult	230	59.90
		Young	154	40.10

2	Sex	Female	240	62.50
		Male	144	37.50

3	Breed	Local	220	57.29
		Cross	164	42.71

4	BCS	Poor	125	32.55
		Medium	109	28.39
		Good	150	39.06

5	Origin	Sululta	96	25.0
		Bishoftu	29	7.0
		Wolaita	58	15.10
		Arba Minch	62	16.15
		Debre Berhan	32	8.33
		Arsi	107	27.86

Total			**384**	**100**

*Note:* The significance of making bold is to give emphasis.

### 3.2. Associated Risk Factors With the Occurrence of *Fasciola* Infection

The present study also assessed the prevalence of ovine fasciolosis in relation to various independent variables of the slaughtered animals. The study analysis result showed the prevalence of ovine fasciolosis (21.43%) was slightly higher in adults than young animals (20%). However, this difference was not statistically significant (*X* ^2^ = 0.12, *p* = 0.734) (Table 2). On the other hand, based on the analysis result, a relatively higher prevalence of ovine fasciolosis was recorded in female sheep (22.5%), while males showed a lower prevalence (17.36%). However, this difference between sexes was not statistically significant (*p* > 0.05). With regard to breed, local sheep showed a markedly higher prevalence of ovine fasciolosis (27.27%) compared to crossbreeds (11.59%). This breed difference was found to be statistically significant (*p* < 0.001). Ovine fasciolosis prevalence was highest in sheep with poor body condition (32.11%), followed by medium (18%) and good (14%) body condition scores (BCSs), and the differences were statistically significant (*p* = 0.001) (Table 2).

**TABLE 2:** Association between ovine fasciolosis prevalence and associated risk factors.

Variables	Category	No. of examined	No of positive	Prevalence (%)	*X* ^2^	*p* value
Age	Young	154	33	21.43		
	Adult	230	46	20.0	0.12	0.734

Sex	Female	240	54	22.5		
	Male	144	25	17.36	1.46	0.23

Breed	Local	220	60	27.27		
	Cross	164	19	11.59	14.2	< 0.001

BCS	Poor	125	40	32.11		
	Medium	109	20	18.35	11.2	0.001
	Good	150	21	14		

Origin	Sululta	96	8	8.33		
	Bishoftu	29	8	27.59		
	Wolaita	58	12	20.69	23.4	< 0.001
	Arba Minch	62	18	29.03		
	Arsi	107	19	17.76		
	Debre Berhan	32	14	43.75		

Ovine fasciolosis prevalence varied markedly across different geographical origins, with infection rates of 8.33%, 27.59%, 20.69%, 29.03%, 43.75%, and 17.76% reported from Sululta, Bishoftu, Wolaita Sodo, Arba Minch, Debre Berhan, and Arsi, respectively. Sheep from Debre Berhan showed the highest infection rate. This variation was statistically significant (*p* < 0.001) (Table 2).

### 3.3. Multivariable Analysis of Associated Factors of Ovine Fasciolosis

Among the assessed risk factors, breed, BCS, and geographical origin were statistically significant in univariable logistic regression and were, therefore, included in the multivariable logistic regression model to check their independent effects using adjusted odds ratios (AOR). Hence, local breed sheep were significantly more likely to be infected with ovine fasciolosis than crossbreeds, with 3.27‐fold higher odds of infection (AOR = 3.27; 95% CI: 1.79–5.98; *p* < 0.001). Similarly, sheep with poor BCS (AOR = 3.70; 95% CI: 1.89–7.25; *p* < 0.001) exhibited markedly increased odds of infection compared with those with good body condition (reference group), whereas sheep with medium BCS was not significantly associated with the occurrence of ovine fasciolosis (AOR = 1.66; 95% CI: 0.82–3.32; *p* = 0.157). In addition, sheep originating from Debre Berhan had significantly higher odds of ovine fasciolosis than those from Sululta (AOR = 5.31; 95% CI: 2.11–13.36; *p* < 0.001) (Table 3). On the contrary, animals originating from Bishoftu showed a significantly lower risk (protective effect) (AOR = 0.39; 95% CI: 0.16–0.97; *p* = 0.04) compared with sheep from Sululta.

**TABLE 3:** Multivariable logistic regression analysis of associated risk factors for ovine fasciolosis.

Associated risk factors		No. of animals tested	Positive samples	*p* value	AOR	95% CI
Breed	Local	220	60	*p* < 0.001	3.27	1.79–5.98
	Cross	164	19	Ref.	—	—

BCS	Poor	125	40	*p* < 0.001	3.70	1.89–7.25
	Medium	109	20	0.157	1.66	0.82–3.32
	Good (Ref)	150	21	Ref.	—	—

Geographical Origin	Debre Berhan	32	14	*p* < 0.001	5.31	2.11–13.36
	Bishoftu	29	8	0.04	0.39	0.16–0.97
	Wolaita	58	12	0.59	1.31	0.48–3.59
	Arba Minch	62	18	0.53	1.31	0.56–3.07
	Arsi	107	19	0.06	2.16	0.98–4.73
	Sululta	96	8	Ref.	—	—

**FIGURE 2:**
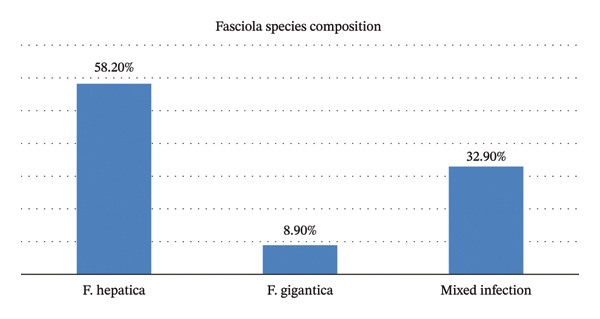
Composition of *Fasciola* species infection.

At postmortem inspection, 46 (58.2%) animals were infected with *F. hepatica*, 7 (8.9%) with *F. gigantica*, and the remaining 26 (32.9%) had mixed infections. The prevalence of postmortem liver examination revealed *F. hepatica* was higher than *F. gigantica* infection and mixed infection (Figure 2). However, the difference between the three categories was not statistically significant (*p* > 0.05).

### 3.4. Financial Analysis of Liver Condemnation

The direct financial loss from liver condemnation because of fasciolosis was computed as the sum of the costs of the condemned livers in sheep slaughtered annually at the abattoir. The present study revealed that 79 livers infected with fasciolosis were condemned.

(3) Annual loss=prevalence×annual slaughtered sheep×unit price of liver.

Given the town’s average liver price of 21 birr, the fasciolosis prevalence reported in this study, and an estimated annual sheep slaughter count for the Addis Ababa abattoir (42,200), the calculated annual economic loss from liver condemnations due to ovine fasciolosis is roughly found to be 182,291 ETB. In brief, based on an annual slaughter rate of 42,200 sheep and an ovine fasciolosis prevalence of 20.57%, an estimated 8681 livers are condemned annually.

## 4. Discussion

Among the animal diseases that affect animal health, fascioliasis is one of the most economically important parasitic diseases of farm animals, particularly in sheep [4, 21]. The prevalence of ovine fasciolosis was 20.57% (95% CI: 16.53%–24.61%) at the Addis Ababa Abattoir Enterprise. This finding is consistent with reports by Dejene et al. [22] in Holeta (26.56%) and Girma et al. [5] in Ejere District, West Shewa (20.92%). Variations in ovine fasciolosis prevalence could be influenced by geographic and climatic factors such as rainfall, altitude, temperature, and humidity, as well as seasonal differences, livestock management practices, community awareness, and the availability of intermediate host snails [23].

The prevalence of ovine fasciolosis in this study was higher than earlier reports of 12.2%–14.8% by Ahmed et al. [6] and Henok et al. [24] in Dire Dawa, Nekemte, the central Awash River basin, and Hirna, respectively. Conversely, it was lower than previous reports of 45.6%–73% by Hubad et al. [25] in Chole Woreda, Michael [26] in Bishoftu, Yilma [27] in Holeta, Tesfaheywet and Negash [28] in Western Hararghe, and Molalegne et al. [29] in Kemisse. Variations in ovine fasciolosis prevalence may be due to ecological and climatic factors such as temperature, moisture, humidity, altitude, soil type, season, and sample size, which affect the distribution of intermediate snail hosts. Additional influences include farmers’ awareness and use of anthelmintics, veterinary service availability, livestock management practices, access to permanent water sources, snail habitat suitability, and ecological changes that impact snail survival [30]. The prevalence of *F. hepatica* and *F. gigantica* in this study was lower than reported by Tadele and Worku [31] at the Gondar abattoir (67.14% *F. hepatica*, 14.1% *F. gigantica*, and 18.77% mixed infection). This variation may be due to differences in environmental conditions, seasonal transmission, livestock management, anthelmintic use, and host factors such as age, immunity, and body condition. Infection with *F. hepatica* was more common as compared with *F. gigantica*. In addition, the geographical origin of sheep slaughtered at Addis Ababa Abattoir Enterprise may also contribute to the observed variation.

Although adult sheep had a slightly higher infection rate than young animals, the difference in ovine fasciolosis prevalence among age groups was not statistically significant (*p* > 0.05). These findings are consistent with those previous reports of Girma et al. [5] and Wondmnew et al. [32], who also reported no significant age‐related differences. However, the results contradict those of Abebe et al. [33]. This observed pattern may be explained by adult sheep tending to graze more widely over larger areas than younger animals and grazing and watering near marshy areas, increasing their chances of exposure to infection [34]. Furthermore, Shanko and Olgira [35] suggested that the higher risk in adults may be related to physiological factors such as stress, pregnancy, lambing, poor nutrition, and concurrent infectious diseases. In this study, fasciolosis prevalence was 22.5% in females and 17.36% in males. The difference between sexes was not statistically significant, which is consistent with previous reports by Chekol and Girma [34], Mohammed [36], Gebreyohannes et al. [37], and Demil et al. [38], who also found no significant sex‐related variation in *Fasciola* infection. This may be attributed to both sexes grazing on the same pastures contaminated with *Fasciola* species.

There was a significant variation in ovine fasciolosis prevalence between breeds (*p* < 0.001), with local sheep being more affected than crossbreeds. This difference is likely due to husbandry practices: local breeds are often raised extensively on communal pastures with waterlogged or marshy areas that favor intermediate *Fasciola* hosts, whereas crossbred sheep are typically managed under semi‐intensive or intensive systems with controlled grazing, limiting exposure to infective stages [30]. Ovine fasciolosis prevalence was significantly higher in sheep with poor body condition (*p* = 0.001) compared to those with medium or good condition, consistent with previous reports from Molalegne et al. [29], Ahmed et al. [6], Henok and Mekonnen [39], and Yemisrach and Mekonnen [40]. This pattern highlights the impact of fasciolosis on body condition, as the disease causes liver damage, reduced feed conversion, anemia, and nutrient loss, leading to weight loss. Consequently, sheep with poor body condition are not only more affected by fasciolosis but also more susceptible to other parasitic infections due to compromised immunity and reduced resilience [41]. Fasciolosis prevalence varied significantly by geographical origin (*p* < 0.001), being highest in sheep from Debre Berhan (43.75%), followed by Arba Minch (29.03%), Bishoftu (27.59%), Wolaita Sodo (20.69%), Arsi (17.76%), and Sululta (8.33%). These results are consistent with reports by Malone et al. [42]. The variation may be due to differences in climate and agroecological conditions, the presence of marshy or irrigated areas, livestock management systems, and altitude, all of which influence the distribution of intermediate snail hosts and fasciolosis transmission [43]. In this study, the protective effects (AOR = 0.39; CI = 0.16–0.97) in Bishoftu town could be unfavorable environmental conditions for snail survival, such as low rainfall, dry grazing areas, better drainage, and improved flock management practices including regular deworming.

Estimating the economic impact of ovine fasciolosis is challenging due to limited prevalence data, difficulty distinguishing direct and indirect losses, and the lack of standardized evaluation methods. In Ethiopia, annual losses have been estimated at around 700 million ETB, including approximately 200 million ETB in productivity losses (2010). The economic impact of ovine fasciolosis varies annually depending on climate, management practices, infection pressure, host immunity, and age and is mainly driven by mortality, treatment and diagnostic costs, liver condemnation, and reduced productivity [44–47]. The annual losses observed in this study exceeded those reported by Berhe et al. [48] in Dessie (25,230 ETB) and Nebiyu et al. [49] in Nekemte (106,536.9 ETB). Direct comparisons of economic losses across studies are limited by differences in slaughter rates, local liver prices, and inflation; our estimate reflects current conditions at this abattoir only.

## 5. Conclusion and Recommendations

The present findings confirm that ovine fasciolosis is a major parasitic disease in the study areas and is causing considerable direct economic losses. Breed, body condition, and geographical origin were identified as associated risk factors. The widespread occurrence of the disease indicates favorable ecological and climatic conditions for the survival of *Fasciola* species and their intermediate hosts, as well as gaps in current control practices. Strategic use of effective flukicidal drugs, particularly in sheep from high‐risk areas such as Debre Berhan before transport to the abattoir, along with improved herd management and further studies on local epidemiology and seasonal dynamics, is essential for sustainable control.

### 5.1. Limitation of the Study

This cross‐sectional study was conducted in a single abattoir, limiting the generalizability of the findings and preventing causal inference. Seasonal variation and management practices were not assessed, and recall bias may have occurred during data collection. In addition, the absence of coprological examination and reliance solely on postmortem findings may have underestimated the true prevalence by missing prepatent, subclinical, or early‐stage infections. Some references were also relatively old due to limited recent studies. Nevertheless, the study provides valuable information on the prevalence and associated risk factors of ovine fasciolosis in the study area.

## Author Contributions

Teshager Dubie: contributed to conception of the research idea, designing and data collection, data analysis, interpretation of data, and writing and editing of the manuscript.

Mimi Kalilo: contributed to data collection, data analysis, interpretation of data, and writing and editing of the manuscript.

Gebremedhin Gebrezgabiher: contributed to data collection, data analysis, interpretation of data, and writing and editing of the manuscript.

## Funding

This research work did not receive any financial support from any institutions or individuals.

## Ethics Statement

Written ethical approval and informed consent for this study were obtained from Samara University’s, Research Ethics Review Committee. Written informed consent was also obtained from the herd owners to take samples from their livestock and for further research use purposes. The reason for this written informed consent is that participants were required for the interview and the individual participant was not subjected to any harm as much as their privacy was kept confidential. Confidentiality of collected data and scientific honesty during the write‐up were considered. These written informed consents were documented.

## Consent

Please see the Ethics Statement.

## Conflicts of Interest

The authors declare no conflicts of interest.

## Data Availability

The data that support the findings of this study are available from the corresponding author upon reasonable request.
